# Compartment-specific importance of glutathione during abiotic and biotic stress

**DOI:** 10.3389/fpls.2014.00566

**Published:** 2014-10-20

**Authors:** Bernd Zechmann

**Affiliations:** Center for Microscopy and Imaging, Baylor University, Waco, TX, USA

**Keywords:** abiotic stress, biotic stress, chloroplasts, drought, glutathione, mitochondria, nuclei, peroxisomes

## Abstract

The tripeptide thiol glutathione (γ-L-glutamyl-L-cysteinyl-glycine) is the most important sulfur containing antioxidant in plants and essential for plant defense against abiotic and biotic stress conditions. It is involved in the detoxification of reactive oxygen species (ROS), redox signaling, the modulation of defense gene expression, and the regulation of enzymatic activities. Even though changes in glutathione contents are well documented in plants and its roles in plant defense are well established, still too little is known about its compartment-specific importance during abiotic and biotic stress conditions. Due to technical advances in the visualization of glutathione and the redox state through microscopical methods some progress was made in the last few years in studying the importance of subcellular glutathione contents during stress conditions in plants. This review summarizes the data available on compartment-specific importance of glutathione in the protection against abiotic and biotic stress conditions such as high light stress, exposure to cadmium, drought, and pathogen attack (*Pseudomonas*, *Botrytis*, tobacco mosaic virus). The data will be discussed in connection with the subcellular accumulation of ROS during these conditions and glutathione synthesis which are both highly compartment specific (e.g., glutathione synthesis takes place in chloroplasts and the cytosol). Thus this review will reveal the compartment-specific importance of glutathione during abiotic and biotic stress conditions.

## INTRODUCTION

**FIGURE 1:**
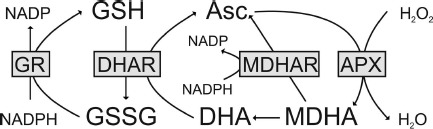
**The ascorbate–glutathione cycle in plants.** Hydrogen peroxide (H_2_O_2_) within the plant cell can be detoxified by ascorbate peroxidase (APX). In this reaction, the reduced form of ascorbate (Asc) is oxidized to monodehydroascorbate (MDHA). MDHA is then either reduced by monodehydroascorbate reductase (MDHAR) to Asc or, since very unstable, reacts to dehydroascorbate (DHA). DHA is reduced by dehydroascorbate reductase (DHAR) to Asc. In this reaction, the reduced form of glutathione (GSH) is oxidized to glutathione disulfide (GSSG). GSSG is then reduced by glutathione reductase (GR) to GSH. The electron acceptor NADP is regenerated during the reduction of MDHA and GSSG by the respective enzymes. Asc and GSH are additional able to detoxify reactive oxygen species by direct chemical interaction. Thus, besides the total ascorbate and glutathione level their redox state (reduced vs. oxidized state) which depends on the activity of the described enzymes (gray boxes) is also very important for successful plant protection.

The subcellular distribution of glutathione in plants is of great importance as this multifunctional metabolite is essential for plant development and growth (Kocsy et al., 2013). It is the most important antioxidant in plants and is a key agent in plant defense against abiotic and biotic stress. It is involved in the detoxification of reactive oxygen species (ROS), either directly through scavenging them or through the ascorbate–glutathione cycle (Figure 1). It is also involved in redox signaling, the modulation of gene expression and the regulation of enzymatic activities (extensively reviewed by Noctor, 2006; Foyer and Noctor, 2009, 2011; Noctor et al., 2012; Kocsy et al., 2013; Schmitt et al., 2014). Additionally, glutathione is involved in the detoxification of xenobiotics, herbicides (Edwards et al., 2005; DeRidder and Goldsbrough, 2006; Cummins et al., 2011; Mohsenzadeh et al., 2011), heavy metals such as cadmium (Zawoznik et al., 2007; Ammar et al., 2008; DalCorso et al., 2008; Ducic et al., 2008; Nocito et al., 2008; Tan et al., 2010; Jozefczak et al., 2012; Koffler et al., 2014b), and protects proteins from oxidation by a process called glutathionylation (Dixon et al., 2005; Hurd et al., 2005a,b; Zaffagnini et al., 2012a,b). Therefore subcellular changes in glutathione contents especially during environmental stress situations provide insights into compartment-specific defense reactions and reflect the occurrence of compartment-specific oxidative stress. Such information can be used as a subcellular stress marker and can be very helpful to clarify the importance of the protective roles of glutathione during stress situations in plants on the cellular level.

Synthesis of glutathione takes place in two adenosine triphosphate (ATP)-depending steps and is highly compartment specific as the enzymes that trigger glutathione synthesis are encoded by single genes which are targeted to either chloroplast and/or the cytosol. In *Arabidopsis*, the first step of glutathione synthesis, the formation of γ-glutamylcysteine (γ-EC) out of glutamate and cysteine catalyzed by γ-glutamylcysteine synthetase (GSH1; also referred to as γ-ECS in some literature, EC 6.3.2.2), takes place in chloroplasts as GSH1 is exclusively targeted to chloroplasts (Wachter et al., 2005). The second step catalyzed by glutathione synthetase (GSH2; also referred to as GSHS in some literature, EC 6.3.2.3.), in which glycine is added to γ-glutamylcysteine to form glutathione, takes place in plastids and the cytosol as GSH2 is targeted to both chloroplasts and the cytosol (Noctor et al., 2002; Sugiyama et al., 2004; Wachter et al., 2005). Nevertheless, it has been shown that restricting the final step of glutathione synthesis to the cytosol is sufficient for normal plant development (Pasternak et al., 2008). These results indicate that chloroplasts export γ-EC to and are able to import glutathione from the cytosol through specific transporters (Maughan et al., 2010) as discussed below. In other plant species, the situation is less clear as GSH1 was also detected in the remaining leaf extract in wheat after the isolation of chloroplasts (Noctor et al., 2002) and as GSH1 is encoded by more than one gene in some plant species (e.g., *Populus trichocarpa*, *Oryza sativa*). Under non-stressed conditions cysteine and subsequently γ-EC are considered to be the rate limiting precursors for glutathione synthesis as it has been shown that both the artificial elevation of cysteine (Gullner et al., 1999; Harms et al., 2000; Bloem et al., 2004, 2007; Zechmann et al., 2007, 2008b) and the overexpression of genes and enzymes involved in cysteine synthesis (Harms et al., 2000; Noji and Saito, 2002; Wirtz and Hell, 2007) increased glutathione contents in plants. The short- and long-term blockage of the first step of glutathione synthesis results in the accumulation of cysteine of up to 300% in some cell compartments also indicating that large amounts of cysteine are used for glutathione synthesis (Koffler et al., 2011). During stress conditions and in the absence of photorespiration glycine can also become the limiting factor for the production of glutathione (Noctor et al., 1997a,b; Zechmann et al., 2008b). The complete absence of glutathione synthesis results in a lethal phenotype (Cairns et al., 2006; Pasternak et al., 2008). Mutants deficient in glutathione synthesis such as the *rml1* mutant which has a single point mutation in the gene that encodes GSH1 develop strong growth defects such as a dwarf phenotype, the lack of a root meristem, short shoots, inflorescence, smaller rosettes, and flowers (Cheng et al., 1995; Vernoux et al., 2000; Cairns et al., 2006). In opposite to *rml1* which shows a reduction of glutathione between 90 and 97% (Vernoux et al., 2000; Cairns et al., 2006) in all cell compartments the *pad2-1* mutant which shows a reduction of glutathione contents of about 80% does not develop a distorted phenotype (Parisy et al., 2007). *pad2-1* mutants are also characterized by a single point mutation of the gene that encodes GSH1 but glutathione contents remain at control levels in mitochondria despite a strong reduction of glutathione in all other cell compartments (Zechmann et al., 2008a; Koffler et al., 2011) which will be further discussed later in this review. Summing up, the ability of plants to synthesize glutathione and the availability of glutathione precursors in glutathione producing organelles are essential for proper plant growth and development and subsequently for defense against abiotic and biotic stress.

Glutathione synthesis is highly compartment specific (e.g., localized in chloroplasts and the cytosol in *Arabidopsis*) but it can be found in different concentrations within the different organelles and accumulates in certain organelles during environmental stress conditions as discussed below. Thus, glutathione transporters must be present in membranes of all organelles in order to facilitate the import and export of glutathione. Whereas the existence of the transport of glutathione through membranes such as the plasma membrane, tonoplast, and the chloroplast envelope is well established (Schneider et al., 1992; Jamai et al., 1996; Foyer et al., 2001; Noctor et al., 2002; Pasternak et al., 2008) the identity and their exact role in glutathione transport still remains unclear especially in plants (Bachhawat et al., 2013). Three low affinity transporters for glutathione have been identified in plants. In *Arabidopsis*, the homologs of the *Plasmodium falciparum* chloroquine resistance transporter (PfCRT) have been described to facilitate the transport of glutathione through the envelope of the chloroplast (Maughan et al., 2010). Three proteins named CLT1, CLT2, and CLT3 were identified to be essential for the transport of glutathione between the chloroplasts and the cytosol (Maughan et al., 2010). Further transporters of glutathione in plants include homologs from the oligopeptide family from yeast. These homologs are mainly associated with the vascular tissue of plants which indicates that they are involved in long distance transport of glutathione rather than transport of glutathione between cell compartments (Koh et al., 2002; Cagnac et al., 2004; Pike et al., 2009). The transport of glutathione conjugates and oxidized glutathione into vacuoles in plants is facilitated by transporters of the ATP-binding cassette (ABC) family (Lu et al., 1998). These transporter might play essential roles in the sequestration of oxidized glutathione in vacuoles in situation of extreme oxidative stress (Queval et al., 2011) as described below.

**FIGURE 2:**
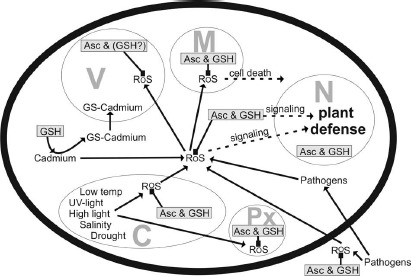
**Compartment-specific production of reactive oxygen species (ROS) induced by different stress conditions and possible detoxification and signaling pathways involving ascorbate (Asc) and glutathione (GSH) and ROS.** C, chloroplast; M, mitochondrium; N, nucleus; Px, peroxisome; V, vacuole.

Glutathione degradation is carried out by γ-glutamyl transferase/transpeptidase (GGT, EC 2.3.2.2) which promotes the cleavage of glutamate from glutathione in vacuoles and the apoplast (Ohkama-Ohtsu et al., 2007a,b; Tolin et al., 2013). In *Arabidopsis*, GGT1 and GGT2 are associated with the cell wall and the plasma membrane, respectively, whereas GGT3 does not seem to have a function, and GGT4 occurs in vacuoles (Ohkama-Ohtsu et al., 2007a,b; Ferretti et al., 2009; Destro et al., 2011; Tolin et al., 2013). A further pathway of glutathione degradation is the removal of glycine from glutathione which is carried out by carboxypeptidase located inside vacuoles (Wolf et al., 1996). Metabolization of the remaining dipeptides is then carried out by dipeptidases. Further pathways could involve the degradation of glutathione by γ-glutamyl-cyclotransferase (EC 2.3.2.4) via 5-oxoproline, which would results in the production of free glutamate (Martin and Slovin, 2000; Ohkama-Ohtsu et al., 2008) or by phytochelatin synthase (also known as glutathione γ-glutamyl-cysteinyltransferase, EC 2.3.2.15) which could facilitate the degradation of glutathione in situations when conjugated glutathione accumulates in the cytosol such as exposure of plants to heavy metals (Grzam et al., 2006; Blum et al., 2007, 2010). As these pathways take place in the cytosol they represent an alternative pathway of glutathione degradation besides the ones in the vacuole and the apoplast (Noctor et al., 2011). As plants depend on the protection of glutathione and as it is also involved in (redox) signaling and the activation of defense genes, its homeostasis in different organs, tissues, and organelles is essential for plant defense and depends on the control of a complex network of metabolic and environmental factors (Noctor, 2006; Foyer and Noctor, 2009, 2011; Miller et al., 2010; Noctor et al., 2012; Szarka et al., 2012). Considering that stress situations affect cell compartments differently (Figure 2) and that glutathione metabolism is highly compartment specific it is essential to investigate subcellular glutathione levels in order to obtain a deeper insight into how glutathione is involved in plant defense.

## METHODS TO DETECT SUBCELLULAR GLUTATHIONE CONTENTS

Despite the importance of glutathione within plant cells its detection on the subcellular level is technically challenging as it can be easily washed out and/or redistributed during sample preparation which is also due to the fact that sample preparation itself can be seen as a stress to the plant sample. Currently, there are different methods available that have been used to study the subcellular distribution of glutathione in plants which can be separated into two major approaches: (1) biochemical measurements after the isolation or fractionation of organelles (Jiménez et al., 1997, 1998; Vanacker et al., 1998a,b,c; Kuźniak and Sklodowska, 2001, 2004, 2005a,b; Ohkama-Ohtsu et al., 2007a,b; Krueger et al., 2009) and (2) microscopical investigations after labeling with specific dyes (Meyer and Fricker, 2000; Meyer et al., 2001; Müller et al., 2005), antibodies (Zechmann et al., 2008a; Zechmann and Müller, 2010) or by using ratiometric redox sensitive green fluorescent protein (GFP; Meyer et al., 2007; Gutscher et al., 2008). All methods have advantages and disadvantages but have given valuable insights into the subcellular distribution of glutathione.

With biochemical methods glutathione was localized and measured after organelle isolation or fractionation in mitochondria, chloroplasts, peroxisomes, the apoplast, and vacuoles of different plant species (Jiménez et al., 1997, 1998; Vanacker et al., 1998a,b,c; Kuźniak and Sklodowska, 2001, 2004, 2005a,b; Ohkama-Ohtsu et al., 2007a,b; Krueger et al., 2009). With these methods it was possible to measure glutathione contents in millimolar concentrations and to differentiate between the reduced and oxidized form which gave valuable insights into the redox state of certain cell compartments during different stress conditions. Nevertheless, these methods face the problem that the individual organelles have to be isolated or fractionated from a large amount of plant samples before glutathione can be measured with high performance liquid chromatography or spectrometrically (Jiménez et al., 1997, 1998; Vanacker et al., 1998a,b,c; Kuźniak and Sklodowska, 2001, 2004, 2005a,b; Ohkama-Ohtsu et al., 2007a,b; Krueger et al., 2009). This can lead to contamination of non-organelle-specific glutathione and because of the lengthy procedure it is unclear how well the obtained results reflect the *in vivo* situation as glutathione can be washed out or redistributed between the organelles (Noctor et al., 2002; Chew et al., 2003; Krueger et al., 2009).

**FIGURE 3:**
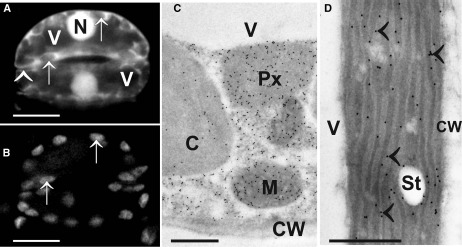
**Images show the typical distribution of glutathione. (A)** Monochlorobimane staining in guard cells of the upper epidermis of tobacco cells in the light microscope. Fluorescence was observed in cytosol and nuclei (N) but not in vacuoles (V) and cell walls (arrowhead). Additionally, no fluorescence could be observed in chloroplasts (arrows in **A** and **B**) which can be best identified when comparing the autofluorescence of chloroplast **(B)** with monochlorobimane staining **(A)**. Transmission electron micrographs show the subcellular distribution of glutathione **(C,D)** in mesophyll cells of leaves from **Arabidopsis** Col-0 plants. Glutathione-specific labeling could be observed in different concentrations in mitochondria (M), chloroplasts (C), peroxisomes (Px) but not in vacuoles (V) and cell walls (CW). Glutathione-specific labeling was observed in the stroma as well as inside the thylakoid lumen (arrowheads) when plants were exposed to high light intensities of 700 µmol m^-2^ s^-1^. Bars in **(A,B)** = 10 µm, **(C,D)** = 0.5 µm.

With light microscopical methods after monochloro- or monobromobimane staining glutathione could be detected in nuclei and the cytosol (Fricker et al., 2000; Meyer and Fricker, 2000; Meyer et al., 2001; Müller et al., 2005). Nevertheless, light microscopical investigations which allow investigations of the *in vivo* situation are limited by the resolution of the light microscope (about 200 nm), by the ability of the antibodies/dyes to infiltrate the different organelles (Müller et al., 2005) and by their specificity to bind with the respective component. Monochlorobimanes, for example, bind to all thiols (not only to the reduced form of glutathione) in cells and do not infiltrate chloroplasts (Hartmann et al., 2003; Müller et al., 2005; Figures 3A,B). Additionally, monochloro- and monobromobimane are toxic to the plant and are transported into the vacuole after complexation with reduced glutathione (Fricker et al., 2000; Meyer and Fricker, 2002). This process can be inhibited by using chemicals that inhibit the transport of glutathione conjugates through the tonoplast such as sodium azide (Fricker et al., 2000). The use of redox sensitive GFP in plants has been proven to overcome some of these problems and has allowed investigations of the glutathione redox potential *in vivo* even in very small cell compartments with fluorescence microscopy (Meyer et al., 2007; Gutscher et al., 2008). As the signal can be targeted specifically to different cell compartments the situation can be even studied in small cell compartments such as the endoplasmic reticulum (ER) which cannot be resolved with the light microscope otherwise (Meyer et al., 2007). Additionally, this method is able to differentiate between the reduced and oxidized form of glutathione and has been used to detect changes in glutathione contents in the cytosol during environmental stress situations (Jubany-Marí et al., 2010; Lim et al., 2014). Nevertheless, *in vivo* investigations with confocallaser scanning or fluorescence microscopes face the problems that only very thin cell layers, tissues, or organs can be investigated and that the sample preparation (e.g., mechanical separation of the epidermis, or other tissues and cells) and investigations with the microscope (exposure to a strong light source, high temperature, lack of oxygen, water stress) can also be seen as a stress source to the sample. Single culture cells are a good alternative but similar to the above-described situation it remains unclear how the situation in single cells, cell types or tissues (e.g., epidermis) correlates with the situation in whole leaves or other tissues. Therefore the labeling results have to be carefully evaluated and the situation in single cells or tissues (e.g., epidermis) cannot always be transferred to the situation in deeper cell layers (e.g., mesophyll, vascular tissue) or the whole plant. Additionally, it was not possible so far with the above-mentioned methods to measure glutathione levels in all cell compartments simultaneously in one experiment.

Another alternative to the above-mentioned techniques is the detection and quantitative evaluation of glutathione after immunogold labeling with computer supported transmission electron microscopy (Zechmann et al., 2008a; Fernandez-Garcia et al., 2009; Zechmann and Müller, 2010; Gao et al., 2012). This method allows the simultaneous detection and quantification of glutathione in all cell compartments of a cell in one experiment (Figures 3C,D). Changes in glutathione contents can be resolved even during stress situations on the subcellular level on a high level of resolution (technical resolution limit of the transmission electron microscope is around 0.2 nm). Thus this method allows the detection and quantification of glutathione in even very small cell compartments (e.g., ER, dictyosomes, membranes) that cannot be resolved by light microscopy (Zechmann et al., 2008a; Zechmann and Müller, 2010). Additionally, it is possible with this method to investigate subcellular glutathione levels also in deeper layers and tissues of the leaves (e.g., mesophyll, vascular tissue). As very little plant material has to be used (sample size is usually around 1 mm^2^) this method can also achieve a very high spatial resolution which is important if one wants to study local events that can induce oxidative stress, e.g., the penetration site of aphids (Zechmann et al., 2009) or fungi (Simon et al., 2013) or wants to look at very small plant material such as pollen grains (Zechmann et al., 2011). Recently this approach was used to calculate glutathione concentrations in millimolar for the different organelles (Koffler et al., 2013). For this purpose, glutathione-specific labeling density was correlated with biochemical analysis of glutathione concentrations in whole organs (see Queval et al., 2011; Koffler et al., 2013 for details). These methods were used to determine subcellular glutathione concentrations (in millimolar) in different leaf areas in older and younger leaves of *Arabidopsis* wild-type plants and during situations of oxidative stress (Queval et al., 2011; Koffler et al., 2013). Highest glutathione concentrations in the center of older leaves were found in mitochondria (14.8 mM), followed by nuclei (6.4 mM), the cytosol (4.5 mM), peroxisomes (4.4 mM), chloroplasts (1.2 mM), and vacuoles (0.08 mM; Table 1).

**Table 1:** **Compartment-specific glutathione contents in leaves of *Arabidopsis* Col-0.**

	Gold particles per µm^2^	Calculated concentrations (mM)
Mitochondria	601	14.9
Chloroplasts	47	1.2
Nuclei	260	6.4
Peroxisomes	179	4.4
Cytosol	181	4.5
Vacuoles	3	0.08
Apoplast	Not detected	Not detected

The amount of glutathione in each compartment was obtained by multiplying the amount of total glutathione measured by HPLC (326 nmol/g fresh weight) with the measured fractional contribution of each compartment to overall gold label. From these values, concentrations were calculated using subcellular volumes estimated in leaf sections of Col-0 (for details, see Koffler et al., 2013).

The main limitations of this method are that the samples have to go through fixation, that the labeling results rely on how well and how close as possible the distribution of glutathione has been preserved to the natural state and on the specificity of the antibodies. Thus, it is impossible to study the distribution of glutathione *in vivo* and the specificity and accuracy of the labeling results have to be carefully and intensively evaluated at the beginning and throughout the use of these methods (Zechmann et al., 2008a, 2011; Zechmann and Müller, 2010). This can be achieved by using different negative controls (e.g., saturation of the antibodies with reduced and oxidized forms of glutathione, using pre-immune serum instead of the antibodies, omission of secondary antibodies), using different fixation methods, and by using mutants that are deficient in or accumulate glutathione (Zechmann et al., 2008a, 2011; Zechmann and Müller, 2010). Unfortunately, the antibody that is currently used for detecting glutathione cannot differentiate between the reduced and oxidized form, thus allowing the compartment-specific detection of the total glutathione status only. Summing up, despite the limitations of these methods they have all contributed toward a deeper understanding of the subcellular distribution of glutathione in plants during abiotic and biotic stress which will be described and discussed in the next sections in connection with their compartment-specific roles during stress conditions.

## SUBCELLULAR RESPONSE OF PLANTS TO ABIOTIC STRESS

### MITOCHONDRIA

The role of glutathione in mitochondria is quite an important one. It seems that high and stable levels of glutathione especially in mitochondria are essential for proper cell development and cell survival especially in situations when the glutathione pool is depleted (Zechmann et al., 2006, 2008a; Zechmann and Müller, 2010). Glutathione contents within mitochondria of leaves and roots of the glutathione-deficient *Arabidopsis thaliana pad 2-1* mutant remained the same as in the wild-type Col-0 whereas glutathione was strongly decreased in all other cell compartments that contained glutathione of up to -91% (Zechmann et al., 2008a). In the glutathione-deficient mutant *rml1*, glutathione labeling was found to be about 96–98% lower in all cell compartments when compared to the wild-type (Zechmann and Müller, 2010). Interestingly, *rml1* showed the strongest reduction of glutathione in mitochondria (-98%). In opposite to *rml1* which forms very short roots (1–2 mm), small shoots and leaves (Cheng et al., 1995; Vernoux et al., 2000), *pad2-1* mutant has a phenotype similar to the wild-type in non-stressed conditions (Parisy et al., 2007). Thus, the preservation of high levels of glutathione in mitochondria in situations of glutathione deficiency seems to be essential for proper plant development. The *rml1* phenotype can also be achieved by treatment of plants with buthionine sulfoximine (BSO), which inhibits glutathione synthesis (Vernoux et al., 2000). Nevertheless, mild BSO-stress did not affect concentrations of glutathione in mitochondria, even though glutathione was reduced in most other cell compartments in roots and leaves (Zechmann et al., 2006). Additionally, short-term excess light stress led to a massive increase of glutathione in mitochondria of the *pad2-1* mutant of up to 900% whereas glutathione contents in all other cell compartments remained unchanged or decreased (Heyneke et al., 2013). These results demonstrate that high and stable levels of glutathione in mitochondria in plants, especially in situations of glutathione deficiency caused by impaired glutathione synthesis induced by BSO or mutations as described above and during abiotic stress, play an important role for the development and growth of plants, thus allowing a phenotype similar to the wild-type.

### CHLOROPLASTS AND PEROXISOMES

Abiotic stress conditions such as excess light, high salinity, and drought (or a combination of all three) represent a unique stress source to the plant that mainly affects metabolic changes in chloroplasts first before other cell compartments are affected (Asada, 2006; Kim et al., 2008; Pfannschmidt et al., 2009). One of the first responses of plants to drought, salt stress and high light conditions is the closure of stomata which limits the gas exchange between the atmosphere and the leaves and decreases the ratio of CO_2_ to O_2_ (Bhargava and Sawant, 2013; Hernández et al., 2013). This situation leads to oxidative stress in illuminated chloroplasts since low levels of CO_2_ in chloroplasts induce disturbances of the Calvin cycle which causes the exhaustion of the primary electron acceptor NADP and the block of the electron transport to NADP. Subsequently electrons will be transferred to O_2_ inducing the production of ROS in chloroplasts (Halliwell and Gutteridge, 1999; Tausz et al., 2004; Asada, 2006; Golan et al., 2006; Krieger-Liszkay et al., 2008; Pospisil, 2012; Schmitt et al., 2014). Additionally, this situation favors photorespiration, which leads to the production of phosphoglycolate. The degradation of this toxic component leads to the production of H_2_O_2_ in peroxisomes (Foyer and Noctor, 2009; Miller et al., 2010; Bhargava and Sawant, 2013; Hernández et al., 2013). Thus it is not surprising that an accumulation of glutathione was observed in chloroplasts and peroxisomes during high light and salt stress conditions (Table 2) indicating an increased need of glutathione in chloroplasts to keep ROS under control. Under excess light conditions glutathione also accumulated inside the thylakoid lumen (Figure 3D) highlighting the importance of high levels of glutathione in chloroplasts for the protection against ROS produced in these cell compartments during excess light conditions (Heyneke et al., 2013). When the scavenging systems in chloroplasts fail to detoxify ROS, which are produced during excess light conditions, plants suffer photobleaching and eventually cell death. Besides toxic effects of ROS which can directly trigger these events by destroying target components within chloroplasts they additionally seem to be actively governed by the plant as the accumulation of ROS in chloroplasts seems to be involved in the regulation of cell death events (op den Camp et al., 2003; Wagner et al., 2004; Doyle et al., 2010; Schmitt et al., 2014). The generation of singlet oxygen in chloroplasts of *flu* mutants within the first minute of illumination correlated with the inhibition of plant growth and the appearance of necrotic lesions (op den Camp et al., 2003). Based on further investigations of gene expression patterns and studies involving the EXECUTER1 protein (which is a highly conserved protein in plastids) the authors came to the conclusion that changes in growth and development of the *flu* mutants after illumination were not caused by direct toxic effects of singlet oxygen but rather reflected its role as a signal initiator that activated several stress-response pathways (op den Camp et al., 2003; Wagner et al., 2004). Additionally, it has been demonstrated in *Arabidopsis* cell cultures under heat treatment that the interplay of antioxidants (especially glutathione and ascorbate) and ROS production in chloroplasts controlled the number of cells that were subject to apoptosis like cell death and the severity of these events (Doyle et al., 2010). Treatment of glutathione and ascorbate under these conditions decreased ROS levels and therefore inhibited necrosis caused by direct damage of ROS in the tissue but increased apoptosis like programed cell death demonstrating the crucial role of ROS in chloroplasts for signaling cell death events (Doyle et al., 2010). Similar results have been obtained during biotic stress conditions where the accumulation of glutathione contents in chloroplasts was insufficient to prevent the accumulation of ROS and resulted in cell death (Großkinsky et al., 2012; Király et al., 2012; Simon et al., 2013). These results support the conclusion that cell death events during stress conditions are not only caused by direct damaging effects of an excess of ROS in chloroplasts but are also indirectly triggered by signaling events induced by ROS in chloroplast (or other cell compartments). Whereas the accumulation of antioxidants in chloroplast under these conditions can reduce direct damaging effects of ROS it cannot prevent the induction of programed cell death.

**Table 2:** **Changes in the subcellular distribution of glutathione in Arabidopsis Col-0 plants exposed to salt stress (treatment of plants with 100 mM NaCl for 3 days; unpublished data) and excess light conditions (700 µmol m^-2^ s^-1^ light for 4 h and 14 days; data according to Heyneke et al., 2013) when compared to plants grown at 150 µmol m^-2^ s^-1^.**

	Chloroplasts (%)	Peroxisomes (%)
High salinity	26***	84**
Excess light (4 h)	27***	32***
Excess light (14 days)	190***	65***

Significant differences were calculated using the Mann—Whitney U-test; ** and ***, respectively, indicate significance at the 0.01 and 0.001 level of confidence. n > 20 for peroxisomes and vacuoles and n > 60 for all other cell structures.

### NUCLEI

The interplay of ROS and glutathione in the nucleus plays an essential role for cell proliferation and subsequently for plant growth and development during abiotic stress (Diaz-Vivancos et al., 2010a,b; Kocsy et al., 2013). Additionally, ROS in nuclei are involved in signaling between nuclei, chloroplasts, and the cytosol especially during stress situations (Galvez-Valdivieso and Mullineaux, 2010; Schmitt et al., 2014). Glutathione contents in nuclei of non-stressed leaves are about double as high than in the cytosol (Koffler et al., 2013) indicating its importance in nuclei. Large amounts of glutathione (up to 80% of the entire cellular pool) have been found to co-localize with nuclear DNA in the early steps of cell proliferation and at the G1 and S phases during the cell cycle (Diaz-Vivancos et al., 2010a). Even the accumulation of H_2_O_2_ in the cytosol could not prevent the sequestration of glutathione into the nucleus indicating that glutathione is transported into the nucleus even in situations of severe oxidative stress in expense of the cytosolic glutathione pool (Diaz-Vivancos et al., 2010a). A similar situation has been found in animal cells where oxidation events early in G1 phase were essential for the activation of signaling events initiating cell proliferation (Menon et al., 2003). It has been demonstrated for plant cells that the depletion of reduced glutathione by BSO inhibited the transition of cells from G1 to S phase (Vernoux et al., 2000). Additionally, the addition of dehydroascorbate and the inhibition of glutathione synthesis by BSO during G1 phase delayed cell division (Potters et al., 2004). On top low levels of ascorbate in the quiescent center of roots seem to be responsible for keeping these cells in the extended G1 state (Kerk and Feldman, 1995). Thus, it seems likely that high levels of glutathione (and ascorbate) in nuclei during G1 phase are an important strategy in order to allow the cell cycle to be continued (Diaz-Vivancos et al., 2010a). If the redox balance of nuclei is altered, DNA could be damaged which could induce mutations and eventually cell death (Diaz-Vivancos et al., 2010b).

### VACUOLES

Recently it has been shown that vacuoles are involved in the protection of plants against ROS during abiotic stress conditions. During high light and drought stress H_2_O_2_ was found to leak from chloroplast and peroxisomes through the cytosol into vacuoles (Heyneke et al., 2013; Koffler et al., 2014a) which might act as a sink for ROS in situations of extreme stress. Nevertheless, glutathione could not be detected in vacuoles during high light and drought stress indicating that glutathione plays a minor role in the detoxification of H_2_O_2_ in this cell compartment. In contrast high levels of ascorbate accumulated in vacuoles under these conditions which could help to reduce phenoxyl radicals (created by oxidation of phenols by H_2_O_2_) and is oxidized to mono- and dehydroascorbic acid which is then transported into the cytosol for reduction (by glutathione through the ascorbate glutathione cycle) to ascorbic acid (Takahama, 2004). The sequestration of oxidized glutathione in vacuoles (10-fold increase in its concentration) seems to be a protective mechanism of the *cat2* mutant in order to avoid proposed negative effects of the accumulation of oxidized glutathione such as lesion formation, dormancy or cell death (Queval et al., 2011). The sequestration of oxidized glutathione and detoxification of H_2_O_2_ in vacuoles during oxidative stress may be involved in the control of cytosolic redox potential and redox state of target molecules and subsequently in the regulation of cell division, differentiation, and defense. Besides being involved in the detoxification of ROS vacuoles also act as a sink for glutathione conjugates withdrawing large amounts of glutathione from the cytoplasm (Fricker et al., 2000; Koffler et al., 2014b). Cadmium, for example, has a high affinity to thiol groups and forms complexes with reduced glutathione. These complexes are then transported into and sequestered in vacuoles (Rauser, 2001; Maksymiec and Krupa, 2006; Semane et al., 2007; Van Belleghem et al., 2007; DalCorso et al., 2008; Jozefczak et al., 2012) which can lead to the withdrawal of glutathione from the cytosol and other organelles if the demand for fresh glutathione cannot be compensated by glutathione synthesis (Koffler et al., 2014b).

## SUBCELLULAR RESPONSE OF PLANTS TO BIOTIC STRESS

### MITOCHONDRIA

The accumulation of ROS in mitochondria during biotic stress conditions is involved in the induction of programed cell death (reviewed by Amirsadeghi et al., 2007; Vianello et al., 2007). The depletion of glutathione in mitochondria favors the accumulation of ROS and thus it is not surprising that in *Nicotiana tabacum* plants infected with an incompatible strain of tobacco mosaic virus (TMV) the decrease of glutathione contents in mitochondria was accompanied with the development of necrotic lesions (Király et al., 2012). Similar effects were observed in *Arabidopsis* plants infected with *Botrytis cinerea*. At the infection site the development of necrosis 48 h post inoculation could be correlated with a strong depletion of glutathione contents in mitochondria, whereas glutathione levels in all other cell compartments remained at control levels (Simon et al., 2013). At this stage and also at later stages of infection the breakdown of the antioxidative defense system in mitochondria could also be correlated with a strong accumulation of H_2_O_2_ in this cell compartment (Simon et al., 2013). In tomato plants infected with *Botrytis cinerea* glutathione contents in mitochondria (besides peroxisomes) were affected the strongest. A strong drop in total glutathione contents accompanied with the accumulation of oxidized glutathione in mitochondria could be observed as early as 48 h post inoculation in this organelle and was accompanied with pathogen induced senescence (Kuźniak and Sklodowska, 2005a). Considering these results the depletion of glutathione contents in mitochondria during pathogen infections seems to favor the accumulation of ROS in this cell compartment and could be the reasons for the induction of programed cell death events. After all it still needs to be clarified if glutathione degradation and the accumulation of oxidized glutathione in mitochondria observed after pathogen infection is actively controlled by the plant or indirectly caused by disturbances in the electron transport chain induced by the invading pathogen.

### CHLOROPLASTS AND PEROXISOMES

Compartment-specific changes in glutathione contents in chloroplasts and peroxisomes seem to be involved in the fine tuning of plant defense against pathogens. During *Botrytis cinerea* and *Pseudomonas syringae* infection in *Arabidopsis*, chloroplasts and peroxisomes could be identified as the hotspots of glutathione accumulation at the infection site at the beginning of the infection whereas the breakdown of the antioxidative system in these two cell compartments at the later stages of infection was correlated with the accumulation of ROS and progress of diseases symptoms (Großkinsky et al., 2012; Simon et al., 2013). Similar results were collected in tomato plants infected with the fungal pathogen *Botrytis cinerea* where the collapse of the antioxidative system in peroxisomes was associated with pathogen induced leaf senescence (Kuźniak and Sklodowska, 2005a). In conclusion, it seems that high levels of glutathione in chloroplast and peroxisomes are essential for a successful defense of plants against fungal and bacterial pathogens. The depletion of glutathione in chloroplast and peroxisomes leads to the accumulation of ROS in the tissue and the progression of symptom development.

### NUCLEI

High levels of glutathione in nuclei play important roles in the protection of sensitive nuclear components (DNA, proteins, etc.) and also in regulating the expression of genes that are involved in the activation of plant defense (Han et al., 2013). Thus, it is not surprising that changes in glutathione contents are commonly observed in nuclei during pathogen attack. In younger zucchini yellow mosaic virus (ZYMV)-infected leaves of *Cucurbita pepo* plants and TMV-infected *Nicotiana tabacum* plants a strong accumulation of glutathione in nuclei was detected after inoculation (Zechmann et al., 2005, 2007; Király et al., 2012). A similar accumulation of glutathione was observed in *Arabidopsis* plants infected with *Pseudomonas syringae* (Großkinsky et al., 2012) and *Botrytis cinerea* at early stages of infection (Simon et al., 2013). These results confirm the importance of high glutathione levels in nuclei during biotic stress. High levels of reduced glutathione in nuclei could serve to protect DNA and redox-sensitive nuclear proteins from oxidation, as well as driving GRX-related processes which would influence the binding of transcription factors which results in adaptations of gene expression patterns (Mou et al., 2003; Gomez et al., 2004; Green et al., 2006; Diaz-Vivancos et al., 2010a,b). On the other hand, it has been demonstrated that the accumulation of glutathione in nuclei accompanied by its depletion in the cytosol subsequently leads to increased glutathione synthesis and the rapid accumulation of glutathione levels in the whole cell (Diaz-Vivancos et al., 2010a,b). Thus, it could be possible that the accumulation of glutathione in nuclei observed during virus, fungal, and bacterial infections in plants (Zechmann et al., 2005, 2007; Großkinsky et al., 2012; Király et al., 2012; Simon et al., 2013) is used as a signal to activate glutathione synthesis in order to increase cellular glutathione contents. This seems very likely as in TMV and ZYMV infected plants (Zechmann et al., 2005; Király et al., 2012), as well as in *Arabidopsis* plants infected with *Pseudomonas syringae* and *Botrytis cinerea* (Großkinsky et al., 2012; Simon et al., 2013) the increase of glutathione in nuclei was followed by a strong accumulation of glutathione in chloroplasts and the cytosol—which are considered to be the primary cell compartments for glutathione synthesis (Wachter et al., 2005).

### APOPLAST

Glutathione contents in the apoplast during non-stressed conditions have been found to be very low or below the level of detection (Vanacker et al., 1998a,b,c, 2000; Zechmann et al., 2008a; Tolin et al., 2013). The reasons therefore can most probably be found in a high degradative activity of GGT1 and GGT2 which degrade glutathione and are located in the cell wall and the plasma membrane, respectively. In *ggt1* knock out mutants levels of glutathione in the apoplast were found to be similar to glutathione contents in plastids (Tolin et al., 2013). These changes were associated with modifications of the proteome that were similar to those found during abiotic and biotic stress conditions. These results indicate that glutathione contents and the redox state in the apoplast are involved in sensing and signaling environmental stress, thus have a key role in the adaption of plants to changing environmental conditions (Tolin et al., 2013). Support for this hypothesis comes from studies during fungal infections in oat and barley plants where a strong accumulation of glutathione was detected in the apoplast which was associated with race- and non-race-specific resistance to *Blumeria graminis* (Vanacker et al., 1998a,b,c, 2000). Decreased amounts of glutathione in the apoplast were found in these plants after infection with a susceptible fungal species where glutathione could not control the accumulation of H_2_O_2_ in the leaves during the hypersensitive response (Vanacker et al., 1998a,b,c, 2000). Such roles of glutathione could not be verified during viral and bacterial infections (Höller et al., 2010; Großkinsky et al., 2012; Király et al., 2012). In conclusion, it seems that glutathione and/or its redox state (which becomes more oxidized during the hypersensitive response) serves important roles in the apoplast in the response to abiotic and biotic stress. Nevertheless, whether glutathione primarily acts as a signaling agent or as an antioxidant in the apoplast still needs to be clarified.

## SUMMARY AND OUTLOOK

Glutathione shows highly compartment-specific changes in plants during abiotic and biotic stress situations, indicating important subcellular roles for plant defense (Figure 2). High levels of glutathione in chloroplasts and peroxisomes seem to be of special importance during abiotic stress situations that negatively interfere with photosynthesis such as high light and salt stress. A drop of glutathione levels in these two cell compartments during pathogen attack could be correlated with the accumulation of ROS and with the development of chlorosis and necrosis. A similar situation was found for mitochondria where high and stable levels of glutathione were found to be essential for proper plant and cell development. The breakdown of the antioxidative system and the accumulation of ROS in mitochondria during pathogen attack seems to be involved in the activation of programed cell death. Vacuoles act as a sink for oxidized glutathione in situations of severe oxidative stress and as a sink for glutathione conjugates, for example, during the exposure of heavy metals. On the other side glutathione does not seem to be directly involved in the detoxification of ROS that diffuse into vacuoles during abiotic stress as observed during high light and drought. The roles of glutathione in the apoplast still have to be clearly dissected but seem to involve sensing and signaling stress conditions rather than the plain detoxification of ROS. In nuclei, glutathione fulfills a dual role during abiotic and biotic stress conditions. On the one site it protects nuclear components against oxidation by ROS and on the other site it is involved in the regulation of genes that are involved in plant defense. In conclusion, glutathione is essential for proper plant development, growth, and defense in the individual cell compartments during abiotic and biotic stress conditions. In order to achieve further progress in this field future research should focus:

(1) on the dissection of the functions of glutathione and its redox state in the apoplast which should help to clarify if its functions are related to sensing and signaling stress rather than simply the detoxification of ROS in this cell compartment,
(2) on the identification and investigation of the physiological relevance of glutathione transporters responsible for the import and export of glutathione from cells, tissues, and especially organelles,
(3) on the evaluation of the relevance of glutathione and antioxidants in vacuoles regarding the detoxification of ROS during environmental stress conditions,
(4) on the correlation, combination and progression of current (and possible new) methods available for the detection of subcellular glutathione in order to achieve a combined measurement of the actual glutathione concentration and the redox state in each cell compartment during abiotic and biotic stress conditions, and finally
(5) on combining this data with changes (i) in the subcellular distribution of ROS, (ii) in the transcription of related genes and (iii) changes in the proteome in order to receive a more detailed picture on the physiological relevance and the interplay of ROS and antioxidants especially glutathione in plants during abiotic and biotic stress.

### Conflict of Interest Statement

The author declares that the research was conducted in the absence of any commercial or financial relationships that could be construed as a potential conflict of interest.
